# Follicle Stimulating Hormone (FSH) as a Predictor of Decreased Oocyte Yield in Patients with Normal Anti-Müllerian Hormone (AMH) and Antral Follicle Count (AFC)

**DOI:** 10.18502/jri.v24i3.13274

**Published:** 2023

**Authors:** Colleen Marie Miller, Ryan Elizabeth Margaret Melikian, Tiffanny LaTrice Jones, Mackenzie Phyllice Purdy, Zaraq Khan, Jessica Lee Bleess, Elizabeth AnNella Stewart, Charles Campbell Coddington, Chandra Camilla Shenoy

**Affiliations:** 1- Division of Reproductive Endocrinology and Infertility, Mayo Clinic, Minnesota, USA; 2- School of Medicine, Wayne State University, Michigan, USA; 3- Conceive Fertility Center, Texas, USA; 4- Kindbody - St. Louis, Missouri, USA; 5- Department of Obstetrics and Gynecology, Atrium Health Carolina’s Medical Center, North Carolina, USA

**Keywords:** Anti-Müllerian hormone, Follicle stimulating hormone, In vitro fertilization, Ovarian reserve

## Abstract

**Background:**

The purpose of the current study was to determine the utility of early follicular phase follicle-stimulating hormone (FSH) testing in patients undergoing in vitro fertilization (IVF).

**Methods:**

This was a retrospective review of patients from 2012 to 2015 at Mayo Clinic in Rochester, Minnesota, USA. Included subjects had a normal anti-Müllerian hormone (AMH) of 1 to 9 *ng/ml* and antral follicle count (AFC) of 10 to 29. Patients were stratified by FSH level when associated estradiol was less than 50 *ng/ml*. In total, 225 patients were categorized into three groups: high FSH (FSH ≥10 *IU/L*; n= 36), normal FSH (>5 *IU/L* and <10 *IU/L*; n=170), and low FSH (FSH ≤5 *IU/L*; n= 19). ANOVA and multiple logistic regression were used for statistical comparisons and for evaluation of the relationships between variables; significance level was set at <0.05.

**Results:**

There were no significant differences in demographics, IVF cycle type, or peak estradiol level between the groups. Patients with a high basal FSH level had a similar clinical pregnancy rate and live birth rate compared to controls and patients with low FSH. High FSH level was associated with decreased follicular development (17 versus 22; p<0.01), oocyte yield (15 versus 18; p=0.02), and embryo yield (8 versus 10; p=0.04) despite higher total doses of gonadotropins.

**Conclusion:**

Patients with normal AMH and AFC levels could be further stratified into lower responders and starting doses of medications can be adjusted based on high basal FSH levels. Therefore, it is suggested to counsel patients on pregnancy outcomes which seem to be quite similar regardless of the FSH level.

## Introduction

As maternal age increases, the likelihood of achieving pregnancy begins to decline as the rate of oocyte aneuploidy increases from a 30% baseline in women aged 35 up to 90% in women in their late 40’s (1). The associated decline in functional ovarian reserve—the number of oocytes available—constitutes the primary challenge associated with achieving conception at older reproductive ages (2). In vitro fertilization (IVF) offers a potential solution to the challenge of decreasing oocyte quality and quantity by allowing clinicians to retrieve a larger number of oocytes, thus increasing the chances of creating competent embryos (1).

How women will respond to gonadotropin stimulation is not always predictable, even among women of similar ages (3). Furthermore, response to gonadotropins does not always correlate with clinical outcomes such as ongoing pregnancy or live birth rate (4). It is imperative to identify patients with a realistic chance of response to treatment due to the significant burdens of undergoing an IVF stimulation including invasiveness, expense, and time commitment (5).

There are three common markers of ovarian reserve used to predict a patient’s response to controlled ovarian hyperstimulation: antral follicle count (AFC), anti-Müllerian hormone (AMH), and early follicular phase follicle-stimulating hormone (FSH) (6). AFC relies on ultrasound to physically count the number of antral follicles in the early follicular phase (7). AMH, produced exclusively by ovarian granulosa cells of preantral follicles, can be measured in the serum to directly predict antral follicle number at any phase of the menstrual cycle (8). Basal FSH is responsible primarily for follicular recruitment and growth and is an indirect and thus less specific marker of measurement of ovarian reserve (8). Each tool has strengths and limitations, and thus these measures are often used in combination. Measurement of follicular phase FSH is often the most inconvenient approach as it must be completed when FSH is at its lowest level (early follicular phase) for accurate results and it may fluctuate between menstrual cycles (8). Patients who have irregular cycles or who are utilizing pre-stimulation hormone suppressors such as oral contraceptive pills cannot rely on FSH testing. The problem becomes even more complicated when the normal FSH level may be falsely low due to negative feedback of the pituitary from an elevated estradiol level (8). Although FSH elevates with a diminishing follicular pool, an increased level is considered a late marker of decreasing fertility and can only predict a poor response to controlled ovarian stimulation at high levels (8, 9). Thus, even a normal FSH cannot rule out a poor ovarian response in some women (9).

Given the limitations associated with the basal FSH test, and the difficulty for women in undergoing early follicular phase testing, there have been divergent viewpoints among experts regarding its relevance in the context of modern IVF cycle planning. While some experts argue that FSH measurement is no longer imperative (10), others caution against relying solely on a single marker of ovarian reserve (11). The purpose of the current study was to assess the efficacy of measuring early follicular phase FSH levels in individuals with normal AMH and AFC results. The underlying hypothesis is that FSH may not serve as a reliable predictor of IVF stimulation outcomes in patients with otherwise normal ovarian reserve. Since most fertility patients fall into this category, the identification of FSH as an unnecessary measure would allow for the elimination of this testing in preliminary IVF planning studies, thereby reducing the need for unnecessary laboratory tests and associated costs. Therefore, the ultimate goal of this investigation was to contribute to the optimization of the preparatory IVF process by evaluating the utility of FSH as a predictive tool in a specific subset of patients with normal ovarian reserve.

## Methods

This study was approved by Mayo Clinic Institutional Review Board (IRB) on February 28th 2017 (Application #17-001369). A retrospective cohort study was designed. Inclusion criteria were patients who underwent their first IVF cycle between 2012 and 2015 at Mayo Clinic in Rochester, Minnesota, USA who had a normal AMH and AFC and a basal FSH value available. Patients were identified from an internal clinical database maintained within the Division of Reproductive Endocrinology and Infertility. AMH was measured by Electrochemiluminescence Immunoassay (ECLIA) and a normal AMH was defined as between 1 *ng/ml* and 9 *ng/ml* in accordance with our laboratory reference values. A normal AFC was defined between 10 and 29 according to our clinic’s reference range of normal. Exclusion criteria included subjects with an early follicular phase estradiol (E2) above 50 *ng/ml* due to likelihood of FSH inaccuracy (10), patients with missing data, and patients who declined access to their electronic medical records.

Baseline patient parameters including age, height, weight, AMH, and AFC levels were obtained from the internal database. IVF cycle parameters collected included: stimulation protocol (co-flare, gonadotropin-releasing hormone agonist [GnRH] or GnRH antagonist), pre-cycle medications (oral contraceptive pills, pre-cycle Estrace, or none), starting dose of FSH and FSH + luteinizing hormone (LH) (150/75, 225/75, 225/150, 300/150), and type of trigger (10,000U human chorionic gonadotropin [hCG], 5,000 *U* hCG, or 4 *mg* leuprolide acetate only). Cycles in which all embryos were frozen and no fresh transfer was performed were categorized according to indication for “freeze-all” stimulation (fertility preservation, oocyte donor usage, plan for pre-implantation genetic testing, and risk of OHSS development). Only outcomes of the patients’ first IVF stimulation and first embryo transfer were analyzed.

Patients were stratified into three groups based on their basal FSH levels: high ≥10 *IU/L*, normal >5 *IU/L* and <10 *IU/L*, and low ≤5 *IU/L*. Elevated FSH of >10 was chosen based on a previous study published by Abdalla and Thum which identified effects on IVF outcomes at FSH of 10.1 *IU/L* and above (11). The primary outcomes were clinical pregnancy rate and live birth rate. Clinical pregnancy rate was determined by presence of fetal cardiac activity on early first trimester ultrasound. Secondary outcomes included number of measurable follicles above 10 *mm* in size on ultrasound, peak estradiol level, number of oocytes retrieved, number of mature oocytes inseminated, and number of normally fertilized embryos. Because of heterogeneity in the embryo stage of transfer and cryopreservation, embryo quality was not included as a secondary outcome. Embryos were transferred between day 2–5 based on clinical factors. Supernumerary embryos were either frozen at the pronuclear stage or blastocyst stage.

Baseline demographics were compared among the three groups using the chi-square test. Outcomes were compared among the three groups using ANOVA. Post-hoc analysis was utilized after the ANOVA for statistically significant findings. The significance level was set at 0.05. Primary outcomes were reported after adjusting for other clinical confounders. Variables in the multivariate analyses were age, body mass index (BMI), AMH, AFC, and basal FSH levels. Statistical analysis was performed using BlueSky v 10.3.1 (BlueSky Statistics LLC, USA). This research study was conducted retrospectively from data obtained for clinical purposes. IRB of Mayo Clinic determined that our study did not need ethical approval. An IRB official waiver of ethical approval was granted from the IRB of Mayo Clinic, Rochester, Minnesota, USA

## Results

A total of 225 subjects were identified and met inclusion criteria. Thirty-six patients (16%) had a high FSH level, 170 (76%) had a normal FSH level, and 19 (8%) had an FSH <5 *ng/dL*. Demographics of the patients can be seen in table 1. There were no significant demographic differences regarding age, BMI, smoking status, AMH, or AFC levels between the three groups.

**Table 1.** Demographics of study participants

**Demographics**	**High FSH (n=36)**	**Normal FSH (n=170)**	**Low FSH (n=19)**	**p-value**
**Age *(years)***	33.3±4.5	32.9±4.6	31.2±5.5	0.26
**BMI *(kg/m^2^)***	24±4	26±6	28±7	0.06
**AMH *(ng/ml)***	2.6±1.5	2.9±1.6	3.2±2.1	0.38
**AFC**	18±5	19±6	20±5	0.19

BMI= Body Mass Index, AMH=Anti-Müllerian Hormone, AFC= Antral Follicle Count.

Data are presented in means +/− standard deviations

Table 2 describes the three groups’ IVF stimulation cycles. The number of subjects who underwent a planned or converted freeze-all cycle was not different between the groups (p=0.14). There were no significant differences in the stimulation protocol and most patients were prescribed combined oral contraceptive pills for down-regulation prior to starting the cycle. The majority of the subjects (n=204) utilized a GnRH antagonist cycle and the cycle selection remained similar regardless of categorization of FSH groups (p=0.84). However, patients with high and low FSH levels were more likely to be started at the highest gonadotropin doses (300 units of FSH and 150 units of combined FSH and LH). Thirty-six percent of high FSH patients and 37% of low FSH patients used this maximum dose protocol as compared to the normal FSH group in which only 19% were prescribed the highest doses (p<0.01).

**Table 2.** IVF stimulation plans among FSH groups

**Cycle descriptions**	**High FSH**	**Normal FSH**	**Low FSH**	**p-value**
**N (%)**	36 (16%)	170 (76%)	19 (8%)	
**Stimulation protocol (n, %)**				
Co-flare	1 (3%)	3 (2%)	0 (0%)	0.84
Full	4 (11%)	12 (7%)	1 (5%)	
Antagonist	31(86%)	155 (91%)	18 (95%)	
**Down-regulation**				
No OCP	2 (6%)	13 (8%)	1 (5%)	0.95
OCP	34 (94%)	154 (92%)	17 (90%)	
Estrace		3 (2%)	1 (5%)	
**Starting dose (FSH only/FSH + LH)**				
150/75	4 (11%)	78 (46%)	10 (53%)	<0.01
225/75	17 (47%)	50 (29%)	2 (11%)	
225/150	2 (6%)	8 (5%)	0 (0%)	
300/150	13 (36%)	33 (19%)	7 (37%)	
**% Started on 300/150 *IU***	13 (36%)	33 (19%)	7 (37%)	<0.01
Freeze-all	11 (31%)	56 (33%)	13 (68%)	0.14
**Freeze-all reason**				
Fertility preservation	4 (36%)	15 (26%)	6 (46%)	0.54
PGT	2 (18%)	17 (30%)	3 (23%)	
OHSS risk	0 (0%)	11 (19%)	1 (8%)	
Other	5 (45%)	14 (25%)	3 (23%)	

IVF= In Vitro Fertilization, FSH= Follicle Stimulating Hormone, n= Number, OCP= Oral Contraceptive Pill, LH= Luteinizing Hormone, Freeze-all= Cycles in which there was no fresh embryo transfer, PGT= Pre-implantation Genetic Testing, OHSS=Ovarian Hyperstimulation Syndrome

Regarding cycle outcomes, the high FSH group received a significantly higher total dose of gonadotropins when compared to the normal and low FSH groups (p<0.01) as seen in table 3. Maximum dose of estradiol during stimulation was similar across the three groups (p=0.36). However, the high FSH group had significantly fewer follicles developed (p<0.01), fewer oocytes retrieved (p=0.02), and fewer embryos created as compared to the normal and low groups (p=0.04). These cycle outcomes did not correlate with a significant clinical difference among patients in their first IVF cycle, as there were no differences in clinical pregnancy (p=0.53) or live birth rates (p=0.69) among the three groups.

**Table 3.** Cycle outcomes across FSH groups

**Outcomes**	**High FSH** **(n=36)**	**Normal FSH** **(n=170)**	**Low FSH** **(n=19)**	**p-value**
**Total gonadotropin dose (*IU*)**	3977	3335	3746	<0.01
**Peak estradiol (*pg/ml*)**	2270	2553	2387	0.36
**No. of follicles recruited**	17±10	22±9	25±10	<0.01
**No. of oocytes retrieved**	15±8	18±9	21±8	0.02
**No. of 2PN zygotes**	8±4	10±6	12±5	0.04
**Clinical pregnancy rate (CPR)**	13/25 (52%)	71/113 (63%)	3/6 (50%)	0.53
**Live birth rate (LBR)**	10/25 (40%)	53/113 (47%)	2/6 (33%)	0.69

No.= number; FSH= follicle stimulating hormone

Multiple logistic regression analysis confirmed there was no significant effect of FSH level on clinical pregnancy and live birth rates. Table 4 summarizes the results of the logistic regression analysis, presenting the odds ratios (OR) and 95% confidence intervals (CI) of each variable included in the final model. The model was adjusted for potential confounding variables, such as age, BMI, ovarian reserve parameters, and baseline characteristics. A normal FSH was not found to be predictive of clinical pregnancy with OR of 1.02 (0.89–1.16) or live birth with OR of 1.00 (0.88–1.15).

**Table 4.** Logistic regression analysis for primary outcomes

	**Clinical pregnancy rate**	**Live birth rate**

	**Adjusted OR (95% CI)**	**Adjusted OR (95% CI)**
**Age**	0.94 (0.85–1.02)	0.92 (0.83–1.00)
**BMI**	0.99 (0.94–1.05)	0.99 (0.94–1.05)
**AMH**	1.2 (0.95–1.53)	1.20 (0.96–1.54)
**AFC**	1.05 (0.98–1.12)	1.05 (0.99–1.12)
**FSH**	1.02 (0.89–1.16)	1.00 (0.88–1.15)

OR= Odds Ratio, CI=Confidence Interval, BMI=Body Mass Index, AMH= Anti-Müllerian Hormone, AFC= Antral Follicle Count, FSH= Basal Follicle Stimulating Hormone

A post hoc power analysis was completed using the data from our study which suggests that the sample should have been comprised of 125 individuals in each group to have adequate power for identification of differences in our primary outcomes. Therefore, the results should be interpreted with caution and a small difference in outcomes between the groups may be present that was not identified in the study.

## Discussion

Among cases with normal AMH and AFC test results, patients with high FSH levels had similar pregnancy rates and live birth rates compared to individuals with normal or low FSH results. However, when examining secondary outcomes, high FSH levels were associated with lower follicular response, oocyte yield, and embryo yield. These differences were seen although these patients were prescribed higher starting medication doses and higher total gonadotropin doses over the course of their IVF cycle. Moreover, only outcomes of patients’ first embryo transfer were available for analysis though clinical pregnancy and live birth rates were not statistically different between the FSH groups. Therefore, there is the possibility that long-term outcomes or cumulative live birth rates could be different in this patient group.

Even though using multiple measures of ovarian reserve testing is common in clinical practice, previous studies have cautioned against utilizing combined testing of ovarian reserve because the tests are often highly correlated with each other, and algorithms that include multiple tests have not proved to be more clinically useful than a single test (12). Conversely, experts have opined that ovarian reserve testing is critical to do via multiple modalities (13). Overall, answering research questions in this area of study is difficult due to differences in threshold values for specific tests. Therefore, our study sought to further investigate this in a clinically meaningful way. A group of patients were identified that were hypothesized to benefit the least from excess testing of ovarian reserve. Basal FSH levels are the most inconvenient tests with wide variability, and several studies have shown FSH testing to be inferior to AMH and AFC testing (14, 15). Thus, an attempt was made to understand if this additional testing could be clinically useful. Unexpectedly, an isolated high FSH level was discovered in 16% of patients who otherwise had normal ovarian reserve parameters. These patients were noted to have clinically meaningful differences in our secondary outcomes. Identifying this specific patient population prior to stimulation allows for medication adjustment and counselling patients on appropriate expectations.

Prior studies have published conflicting results when examining the value of basal FSH testing as a predictor for pregnancy after IVF. One study of 101 patients found that 40-year-old women with normal FSH levels <15 *IU/L* had better implantation rates per embryo and higher ongoing pregnancy rates versus women 40 years old or younger with elevated FSH levels >15 *IU/L* (16). However, Chuang et al. analyzed 1,045 women during their first IVF cycle and found that while age and basal FSH predicted poorer laboratory outcomes, only age was associated with decreased implantation and pregnancy rate (17). Daney de Marcillac et al. analyzed the mean number of oocytes in 1803 cycles with respect to both AMH and FSH and included a group with normal AMH and elevated FSH; this group performed similarly compared to the normal AMH and normal FSH group (18). In our smaller study, early follicular phase FSH was not a valuable predictor of pregnancy or live birth rate in the context of other normal ovarian reserve parameters; however, a difference in retrieved oocytes was observed. Overall, it can be hypothesized that because patients with a normal FSH obtained more oocytes and embryos, cumulative pregnancy rate outcomes including future frozen embryo transfers could have additional implications. Despite the smaller size of this study, it adds to the literature because of its unique focus on a subset of patients with normal AMH and AFC levels.

It is also important to consider that neither FSH nor AMH and AFC levels were predictive of live birth in this study. This is likely because of a homogenized patient population who had an inherently good prognosis (normal AMH and AFC levels). Larger studies are required to understand the correlation between these different measures of ovarian reserve. Interestingly, many researchers who conducted larger studies that have sought to model IVF outcomes neglected to include FSH testing as a parameter (19, 20). This may be because of the difficulties in obtaining this value for patients prior to starting IVF. It is more than likely the various ovarian reserve tests are cumulative rather than redundant and testing all markers of ovarian reserve together will provide the most detailed information (21).

To the best of our knowledge, this is the first study to examine the utility of basal FSH testing among patients with normal AMH and AFC levels prior to an IVF stimulation. All patients underwent stimulation at a single academic practice, thereby minimizing inter-clinic variability. However, the main limitations of this study are the limited follow up beyond the first embryo transfer and the arbitrary cut-offs of “normal” for AMH and AFC. Other clinics and laboratories may use other definitions for a normal AMH value or antral follicle count. Additionally, alternative studies could have been found in which authors have chosen different cut-offs for the low and high FSH groups. Another limitation of the study was the sample size. The number of patients was not sufficient to confidently identify differences in primary outcomes.

## Conclusion

In this retrospective review, high FSH levels were associated with decreased follicular development, oocyte yield, and embryo development in patients with a normal AMH and AFC. This occurred despite receiving higher total doses of gonadotropins by patients. Although pregnancy rates and live birth rate (LBR) were not statistically different in the first IVF transfer cycle, the differences in number of embryos created may have long-term implications in cumulative pregnancy rates for these patients. FSH testing remains important to help providers plan IVF stimulation dosing as well as counsel patients on anticipated outcomes.
